# Ignoring Adjuvant Toxicity Falsifies the Safety Profile of Commercial Pesticides

**DOI:** 10.3389/fpubh.2017.00361

**Published:** 2018-01-22

**Authors:** Robin Mesnage, Michael N. Antoniou

**Affiliations:** ^1^Gene Expression and Therapy Group, Department of Medical and Molecular Genetics, School of Basic & Medical Biosciences, King’s College London, Guy’s Hospital, London, United Kingdom

**Keywords:** pesticides, adjuvants, toxicity tests, risk assessment, endocrine disruptors, surfactants

## Abstract

Commercial formulations of pesticides are invariably not single ingredients. Instead they are cocktails of chemicals, composed of a designated pesticidal “active principle” and “other ingredients,” with the latter collectively also known as “adjuvants.” These include surfactants, antifoaming agents, dyes, etc. Some adjuvants are added to influence the absorption and stability of the active principle and thus promote its pesticidal action. Currently, the health risk assessment of pesticides in the European Union and in the United States focuses almost exclusively on the stated active principle. Nonetheless, adjuvants can also be toxic in their own right with numerous negative health effects having been reported in humans and on the environment. Despite the known toxicity of adjuvants, they are regulated differently from active principles, with their toxic effects being generally ignored. Adjuvants are not subject to an acceptable daily intake, and they are not included in the health risk assessment of dietary exposures to pesticide residues. Here, we illustrate this gap in risk assessment by reference to glyphosate, the most used pesticide active ingredient. We also investigate the case of neonicotinoid insecticides, which are strongly suspected to be involved in bee and bumblebee colony collapse disorder. Authors of studies sometimes use the name of the active principle (for example glyphosate) when they are testing a commercial formulation containing multiple (active principle plus adjuvant) ingredients. This results in confusion in the scientific literature and within regulatory circles and leads to a misrepresentation of the safety profile of commercial pesticides. Urgent action is needed to lift the veil on the presence of adjuvants in food and human bodily fluids, as well as in the environment (such as in air, water, and soil) and to characterize their toxicological properties. This must be accompanied by regulatory precautionary measures to protect the environment and general human population from some toxic adjuvants that are currently missing from risk assessments.

## Introduction

Human tissues are impregnated with chemicals used in commercial formulations of pesticides (1), which are collectively referred to as “pesticide residues.” This is the conclusion reached by governmental biomonitoring programs, raising questions about long-term health effects of a daily exposure to pesticide residue mixtures. These residues generally arise from the ingestion of contaminated agricultural crops sprayed with herbicides, insecticides, or fungicides. However, pesticide formulations are not only used in agriculture but also in other sectors (public or private parks, gardens, along roads and railway tracks, etc.), providing additional routes of exposure. Some recent studies indicate that the domestic use of insecticides (e.g., repellents, acaricides), fungicides (as furniture treatments), or herbicides (for domestic gardening) could be a major source of human exposure (2).

Recent toxicological studies indicate that some pesticides cause cancer and affect the central nervous system, or even interfere with (neuro)endocrine functions, resulting in metabolic and reproductive defects (3). However, regulatory studies have been often unsuccessful at predicting the toxic effects of these pesticides based on the multiple tests conducted before commercial approval (4). A number of pesticides were thus banned after decades of use because certain unexpected health effects occurred in human populations after major contamination accidents or after decades of exposure as highlighted by epidemiological studies.

The different ingredients present in a given pesticide formulation can be regulated differently, and some are even unregulated because they are considered to be “inert” additives, in the sense that are devoid of pesticide activity. However, studies have revealed that these supposedly “inert” diluents can be more toxic than the regulated active pesticide principles (5). We describe here how unregulated chemicals present in commercial formulations of pesticides could provide a missing link between observed negative health outcomes and pesticide exposure.

## Components of Commercial Pesticide Formulations

**Figure 1:**
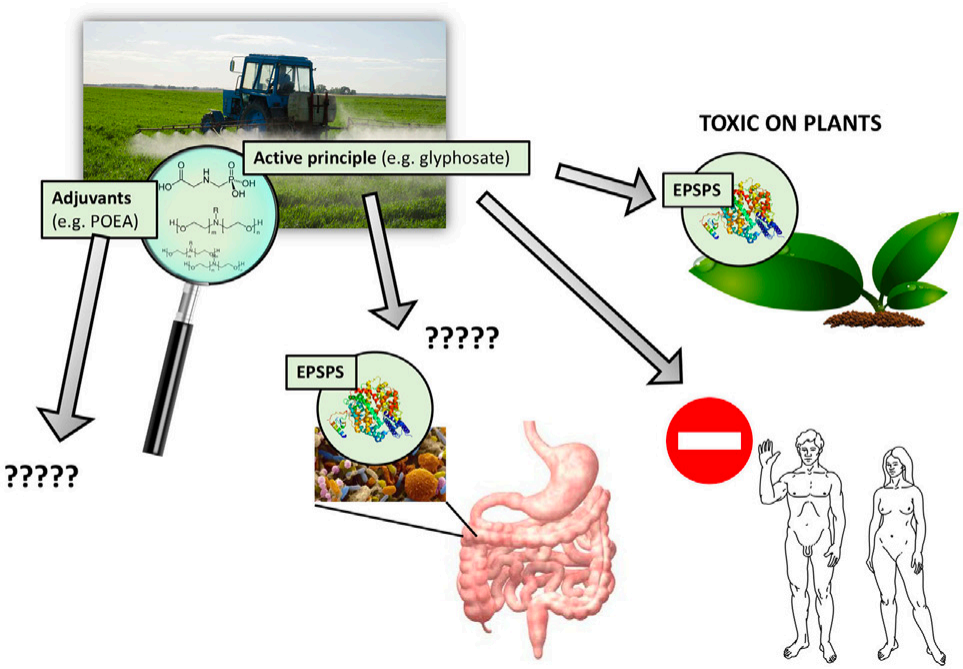
Pesticides are never used alone but in combination with adjuvants. Agricultural preparations of pesticides include adjuvants mixed with an active principle to increase toxic effects. For glyphosate-based herbicides, the active principle primarily targets the EPSPS enzyme but needs adjuvants such as polyethoxylated tallow amine to penetrate into plant tissues and cells. These adjuvants can also be toxic in their own right; numerous toxic effects have been reported in humans and the environment. However, adjuvants are regulated differently than active principles, and their long-term toxic effects are generally ignored and thus missing from pesticide risk assessment procedures.

Commercial formulations of pesticides are invariably not single ingredients. Instead they are cocktails of chemicals, composed of an active principle and “other ingredients.” Sometimes also called “inerts,” “co-formulants,” or “adjuvants,” these other ingredients are specifically added to influence the absorption and stability of the active principle and thus promote its pesticidal action (Figure 1) (6). These compounds are generally included as co-formulants in commercial formulations of pesticides with an active ingredient, but they are also sold and used separately as adjuvants and added during the preparation of the agriculturally applied pesticide mixture. Their use is expected to increase. The market for agricultural adjuvants, valued at USD 2.51 billion in 2015, is projected to increase by 5.8% to reach USD 3.18 billion by 2019 (7).

The major adjuvants are surfactants. The most common are non-ionic surfactants such as ethoxylated alkylphenols. Surfactants are added to pesticides to form micelles, which increase the solubility of the active ingredient and protect it from degradation. This increases the half-life of the active ingredient and improves its pesticidal activity (8). For instance, penetration of the active ingredient diclofop-methyl into maize leaves was increased by seed oil additives (9). Experiments were conducted to estimate the effect of an addition of adjuvants (oil, surfactant, and multicomponents) on the behavior of the herbicide active ingredient metazachlor in soil. The adjuvants increased the half-life of metazachlor and slowed down its leaching and degradation in soil (10). Adjuvants used in pesticide formulations also include spreaders, stickers, (anti)foaming agents, dyes, and drift retardants that modify the physicochemical properties of the spray mixture (Table 1) (11). For example, diesel fuel and kerosene are used as antifoam agents to reduce foam formation in the tank during spray mixture preparation (12). Drift retardants such as polyvinyl and polyisobutylene polymers increase spray droplet size and thus reduce the number of small droplets that are susceptible to be carried and spread by the wind (9). Solvents can also be used to increase pesticide mobility. Heptanol, octanol, and nonanol were able to increase the mobility of 2,4-D in cell membranes of bitter orange leaves by 25- to 30-fold (9). The composition of adjuvants depends on the physicochemical properties of the active principle, as well as on the types of formulation (emulsifiable concentrate, wettable powder, solution, granules, etc.).

**Table 1:** Overview of adjuvants used in pesticide formulations.

Adjuvant type	Example
Penetration agents	Petroleum or mineral oils, vegetable oils, organosilicon

Oder masking agent	1-octanal

Dyes	fd&c blue no. 1, fd&c red no. 40

Preservatives	Hexamethylenetetramine, potassium benzoate, sorbic acid

Stabilizer	Diisopropanolamine, hydroxyethylidene diphosphonic acid, silver nitrate

Diluents	Aluminum hydroxide

Surfactants	Anionic: alkylbenzene sulfonates, sodium laureth sulfate, soap
	Cationic: dioctadecyldimethylammonium chloride
	Amphoteric: cocamidopropyl betaine, cocamidopropyl hydroxysultaine
	Non-ionic: alkoxylated alcohol, ethoxylated alcohol, nonylphenol ethoxylate, tallow amine ethoxylate, alkyl polyoxyethylene ether

Emulsifiers	Alkanoic and alkenoic acids, monoesters and diesters of α-hydro-ω-hydroxypoly (oxyethylene), glyceryl monostearate, sodium metasilicate

Propellant	1,1-Difluoroethane, butane, propane

Solvents	*N*-methyl-2-pyrrolidone, polychloromethanes, chlorinated volatile organic compounds, xylene, isopropanol

Antifoaming agent	silicones (e.g., dimethylpolysiloxane), fatty acids

Carriers	Biochar, cyanobacteria, clay minerals, siliceous minerals, zinc-layered hydroxide, polymeric materials such as chitosan, lignin, and poly(ethylene) glycol

*This non-exhaustive list presents compounds grouped by category that are classically used as adjuvants in commercial pesticide formulations. Some of these molecules can have dual roles. For instance, surfactants (wetters) are also used as plant penetration agents. Compiled from Ref. (13–16)*.

One should also note that adjuvants do not only increase the penetration of the active pesticide ingredient into plants but also of the skin of those exposed, as shown in the comparison of the dermal penetration of atrazine, alachlor, and trifluralin to their commercial formulations Aatrex, Lasso, and Treflan (17).

## Adjuvants Can be More Toxic Than Active Principles

It is recognized that adjuvants alone can have phytotoxic activities (9). However, adjuvants are generally stated to be inert for non-target species. Historically, the classification of a compound as either active or inert in pesticides was first introduced by the Federal Insecticide, Fungicide, and Rodenticide Act in 1947 in the United States (18). A pesticide compound is considered to be active when intentionally added to be toxic to target species. All others are defined as inert ingredients, although this does not exclude their own toxicity, including on non-target species. However, the term “inert” was still understood as meaning biologically inactive 50 years later and, therefore safe, as shown by a consumer survey conducted by the US Environment Protection Agency (19). This is why the US EPA started to use the term “other ingredients” to describe adjuvant mixtures.

In this review, we have focused on the co-formulants included in the commercial formulations of glyphosate-based herbicides (5), in particular the polyethoxylated tallow amines (POEAs), which we consider as a representative good model system because they are the most used pesticides worldwide (20) (Figure 1). We also investigate the case of neonicotinoid insecticides, which are strongly suspected to be involved in bee and bumblebee colony collapse disorder.

### Example 1: Glyphosate-Based Herbicides

A total of 750 different formulations of glyphosate-based herbicides are marketed worldwide (21). Indeed, each name of a given formulation represents a different mixture of active principle and co-formulants (Table 1). As a result of the variability in co-formulants, and since most of them are not compulsorily declared, the effects of pesticides are complex and combinatorial. The literature is quite heterogeneous because these co-formulants vary between commercial pesticide formulations and thus have different and/or additive side effects between themselves and with glyphosate. In fact, this causes confusion in the scientific community, with authors not always declaring the formulation that they tested (22). Authors even sometimes confuse commercial formulations with the active ingredient; they state that “glyphosate” was used when in reality they employed a formulation in their studies. The problem of reproducibility and consistency in the results of toxicological studies (23) could be partly due to the fact that comparisons are performed between different formulations. Some studies even compare formulations and glyphosate alone treating the two as equivalent and therefore ignoring the effects of adjuvants in the former (22). This adds yet further confusion and questions over the reliability of the data obtained. For example, a recent study investigating effects of a Roundup formulation on damselfly larvae concluded that “the toxicity of Roundup cannot be fully attributed to the surfactant POEA” and that “Roundup^®^ […] contains POEA as surfactant” (24). This is not accurate as not all Roundup formulations contains a POEA surfactant and that the manufacturer company Monsanto has moved away from the use of POEA-based surfactants in their latest generation of Roundup formulations (5). The authors of this study do not indicate which commercial formulation of Roundup they have tested on damselfly larvae, and thus it is unknown if POEA was present. Therefore, although they present interesting results on the toxicity of Roundup on an environmental toxicity indicator organism, at the same time they bring confusion to the field by concluding that their study “confirms the toxicity of the surfactant POEA.”

Séralini and colleagues have conducted the most extensive study on the composition and toxicity of the different ingredients that constitute glyphosate-based herbicides. They have compared the toxicity of different brands of glyphosate-based herbicides in tissue culture cell assays and showed that several commercial formulations were up to 1,000 times more toxic than glyphosate alone, the regulated active ingredient (5). In addition, adjuvant mixtures generally contain several ingredients, and these can sometimes be mixtures themselves. For example, POEAs are mixtures of diethoxylates of tallow amines with different toxicological properties (25), which are characterized by their oxide/tallow amine ratio. The toxicity of POEA increases as the tallow amine chain is shortened. The most commonly used POEA is POE (15) tallow amine (POE-15), which was used in the first formulations of glyphosate commercialized under the trade name “Roundup.” By using cell culture model systems, Séralini and colleagues demonstrated that the toxicity of glyphosate-based formulations was proportional to their concentration of POE-15 or other ethoxylated surfactants (5). The formulations that did not contain ethoxylated surfactants were approximately 100 times less toxic to human cells. It was quite a surprising finding to see that the toxicity of two formulations of the same active ingredient could differ by a factor of 100. Thus, the consumer could buy one or another glyphosate-based herbicide formulation without being aware of this difference in toxicity.

The study by Séralini and colleagues based on tissue culture cell lines clearly has its limitations, including exacerbating the observed differential toxicity profiles of the formulations tested. However, the findings of greater toxicity of commercial formulations over glyphosate alone *in vitro* have been replicated *in vivo* in laboratory animals (26), other animal model systems such as sea urchins (27), microorganisms (bacteria, microalgae, protozoa), and crustaceans (28). A more recent study on two life stages of the Pacific oyster shows that POEA-based adjuvants can be very toxic to embryonal and larval development (EC_50_, 262 µg/L) (29). Metamorphosis tests revealed that although EC_50_ values exceeded 100,000 µg/L for glyphosate and its metabolite aminomethylphosphonic acid, they were as low as ~6,000 μg/L for some commercial formulations (30). Studies have also revealed that some ethoxylated adjuvants can be endocrine disruptors at lower non-toxic concentrations. Recently, it has been reported that POEA-based adjuvants promote triglyceride accumulation in 3T3-L1 adipocytes at concentrations from 0.1 to 10 µM (31). This is in contrast to glyphosate alone, which did not promote lipid accumulation in this same adipocyte cell line (Mesnage and Antoniou, unpublished results). Another study has shown that ethoxylated adjuvants can inhibit aromatase activity disrupting estrogen–androgen balance (32).

A comparison of the effects of a glyphosate-based herbicide and glyphosate at an equivalent concentration of 25 mg/kg/d on the composition and metabolism of the gut microbiome in Sprague-Dawley rats found that the commercial formulation but not glyphosate alone affected the numbers of observed species in both the cecum and the colon (33). Although glyphosate has been patented as an antiparasitic agent and suggested to be a bacterial antibiotic (US patent number: US7771736 B2), it is likely that the effects of glyphosate-based herbicides on the gut microbiome could be due to the damaging properties of surfactants present in the adjuvant mixture on the integrity of the intestinal epithelium. In support of this possibility is the observation that emulsifiers have been shown to alter gut microbiome composition in mice by sweeping the lining of the gut, which consequently gave rise to colitis and metabolic syndrome (34).

More recently, we have shown that the chronic (2 years) administration of a glyphosate-based herbicide (Roundup) induced liver toxic effects in rats at an environmental concentration and daily intake of active ingredient was declared safe by regulatory agencies worldwide (35, 36). However, further research is required to elucidate whether the glyphosate, the adjuvants, or the combination of the two is at the basis of the observed kidney and especially liver toxicity seen in these animals. It is difficult to attribute the toxicity of a commercial formulation to a given ingredient if they are not tested in parallel in an experiment. Glyphosate-based herbicides can not only contain POEA but also contain multiple adjuvants having intrinsic toxicological properties. These formulations can also include methylchloroisothiazolinone having allergenic properties, light aromatic petroleum distillates having liver toxic effects, or sodium o-phenylphenate considered as possibly carcinogenic to humans (22).

This and other work has led the European Commission to recommend a ban on the use of POEA-type adjuvants in glyphosate-based herbicide products. Although this can be seen as a positive step forward for public health, this does not exclude the use of POEA in other non-glyphosate-based products. For example, in France, 126 formulations of glyphosate were removed from the market in July 2016, but other POEA-containing pesticides can still be bought. In addition, French farmers can still source POEA as a separate adjuvant mixture (product name Regain, authorization 9300433) to mix with a glyphosate formulation in the spray tank (https://ephy.anses.fr/adjuvant/regain). POEA is also still authorized as a co-formulant in pesticide formulations containing other ingredients such as 2,4-D. Thus, farmers and the general public can still readily be exposed to POEA despite it being banned in glyphosate-based herbicides.

Furthermore, the finding that POEA is widely found in fields in the United States where glyphosate-based herbicides are applied (37) raises concerns that this and other classes of pesticide adjuvants may be entering the food and feed chain undetected, with as yet unknown health consequences.

### Example 2: Neonicotinoid Insecticide Formulations

Neonicotinoids are synthetic insecticides targeting nicotinic acetylcholine receptors in the central nervous system of insects. Their intensive use in agriculture has been associated with a wide range of toxic effects on non-target organisms leading to, for example, colony collapse disorders in social insects such as honeybees and bumblebees (38, 39). Another well-documented case of adjuvant toxicity of note is the organosilicon surfactants used in some neonicotinoid insecticide formulations. Organosilicon surfactants are a class of silicon-based polymers used to modify the surface tension of plant cells and insect cuticles to increase the penetration of pesticide active ingredients and can constitute up to 2% of the spray tank mix.

A series of publications by Mullin and colleagues have revealed their profound effects on honeybees (40). This ranged from acute toxic effects to olfactory learning impairments (41). These authors analyzed the contamination of honey, pollen, or beeswax by trisiloxane surfactants and found it was present in every beeswax (up to 390 ng/g) and 60% of the pollen (39 ng/g) samples (42). They also studied pesticide applications in almond orchards in California and showed that the use of organosilicon surfactants increases during the flowering season (43). This is the period when two-thirds of US honey bee colonies are present. This suggests that the neglect of pesticide tank mixture-derived toxicities could account for the knowledge gap in the cause of bee colony collapse syndrome. Although some studies suggest that organosilicon surfactants are among the least toxic surfactants to bacteria compared to ethoxylated surfactants such as POEA (13), the situation is very different for honey bees as a concentration of 100 pM of an organosilicon surfactant induced 60–100% mortality when the POEA had no effect at this concentration (40). The effects of co-formulants can also be more indirect as a study has even shown that adjuvants can potentiate viral pathogenicity in honey bee larvae (44).

Another more recent study showed that a co-formulant used in insect growth regulators (*N*-methyl-2-pyrrolidone) can have adverse effects on honey bee reproduction and development (45). The authors of this study also revealed that the adjuvant co-formulants can also have an unexpected persistence. A growth chamber experiment showed that *N*-methyl-2-pyrrolidone can persist in pollen for up to 7 days at concentrations reaching 69.3 ppm. As *N*-methyl-2-pyrrolidone is widely used and can also be present in neonicotinoid formulations (such as Confidor), it has the potential to negatively affect the well-being of wild bee populations *via* the use of this class of pesticides. In addition, the *N*-methyl-2-pyrrolidone is a developmental toxicant and caused malformations such as incomplete ossification of the skull in rats, suggesting toxicity to other non-target organisms including mammals (46).

The differential effects between neonicotinoid-based formulations and their active principles have been confirmed on other invertebrates. The toxicity of Apache 50 WG^®^ formulation was found to be 46.5 times more toxic than could be explained by its active ingredient clothianidin alone on *Daphnia magna* (47). Contrastingly, preparations of Calypso 480SC^®^ (containing thiacloprid) and Actara 240 SC^®^ (thiamethoxam) were two to three times less toxic than their respective active insecticide principles.

## Regulatory Guidance Values for Pesticides Can be Miscalculated by Ignoring Adjuvant Toxicity

The identity of these “inert” additional adjuvant ingredients in pesticide formulations is frequently undisclosed as they are considered to be confidential commercial information. The US EPA has a list of compounds authorized in pesticide formulations, but it does not require the registration or labeling of spray adjuvants. As they are proffered as “inert,” they are ignored by regulatory agencies in the determination of acceptable daily intake (ADI) levels, a threshold value of exposure in terms of a unit of weight, usually milligrams, per kilogram body weight per day of ingestion to a “pesticide.” A dose at or below the ADI is deemed unlikely to result in any negative health effects.

In the field of public health, a large paradigm shift took place in the middle of the twentieth century in the form of a growing awareness of the health risks associated with chemical pollutant exposures arising from food and feed. This resulted in the implementation of ADIs in 1954 by the US FDA (48). The ADI for a given pesticide active ingredient is derived from laboratory animal experiments performed by industry in support of regulatory approval. The objective of these experiments is to ascertain the dose of the chemical that results in a no observed adverse effect in the animals. Once this “no observed adverse effect level” is defined for the chemical in question, it is divided by a predetermined value to account for uncertainty factors and thus provide a greater margin of safety. Typically, a factor of 10 is applied for animal to human extrapolation and another factor of 10 for interindividual variability in the human population. Testing of whole pesticide formulations instead of just active ingredients alone would constitute a precautionary approach ensuring that the calculated guidance value (ADI) is valid for the worst case exposure scenario. Such chronic tests in animals are also used to predict other combined effects with different compounds, such as the estimation of the hazard index. However, the current risk management includes many safety guidance values in addition to the ones described. Considering adjuvants as inert compromises the validity of some pesticide environmental risk indicators, for instance the Groundwater Ubiquity Score or the Environmental Impact Quotient (49). It is established that the half-life of pesticide active ingredients in soil is extended by the presence of adjuvants as has been demonstrated for chloridazon (50). A study investigating the leaching of four pesticide formulations (azoxystrobin, propyzamide, triadimenol, and cyproconazole) through a sandy loam soil found that leaching was greater than was the case with their respective active ingredients alone (51).

Neglecting adjuvants may also impact the validity of the authorized maximum residue level (MRL). The MRL is supposed to ensure the safety of food/feed consumption. These chemical residue limits represent the maximum expected when applying a pesticide according to good agricultural practice. However, livestock feeding studies are generally performed with active ingredients alone, which therefore ignore the mixture effects from the adjuvants that are also consumed.

Issues regarding the differential and combined toxic effects of pesticide ingredients can also be considered from the perspective of chemical mixture toxicology. The understanding of combined effects of chemical mixtures is a massive challenge for toxicology as humans and the environment as a whole are exposed to a huge number of chemical pollutants. To simplify this problem, a common strategy is to prioritize the study of chemical mixtures, which are frequently found together. This is typically the case of pesticide commercial formulations. These mixtures can be deconstructed to either predict the toxicity of the mixture from the toxicity of their constituents or the whole mixture can be tested directly as it is found in the environment. According to scientific committees of the EU (adopted by EFSA), the whole-mixture approach is recommended for any unidentified materials in the mixture and for any interactions among mixture components (52). This is typically the case of pesticides, which are nevertheless evaluated as single ingredients. A similar approach is suggested in the US by the Food Quality Protection Act of 1996, which has been implemented for pesticide assessment to reflect real-life scenarios (53). This includes recommendations to consider aggregate risk from exposure to some pesticide ingredients. The first, immediate exposure to a mixture in a typical general public and occupational context is that of active principles and adjuvants sold and used as pesticides. Following current strategies recommended by regulatory bodies to estimate the risk arising from the combined exposures to pesticide residues, would thus in principle lead to the consideration of mixture effects arising within the components of pesticide commercial formulations.

## Exposures to Adjuvants Have Health Effects on Human Populations

Tests conducted for regulatory purposes are performed with the industry-stated active principle alone. This can be a valid approach to establish a reference for active principles, but does not represent the toxicological properties of the commercialized products as used in both agricultural and urban/domestic environments. Chronic effects on mammals of complete commercial formulations of pesticides are never tested. Only short-term acute toxic effects are studied, which appears to be based on the promise that a combined exposure to the ingredients of these formulations is only likely to occur in exceptional circumstances such as intentional ingestion in suicide attempts and accidental exposure due to mishandling. Nonetheless, exposure to environmental levels of some of these adjuvant mixtures has been associated with chronic human disease. For example, in epidemiological studies of farming populations, people exposed to supposedly inert ingredients such as solvents or petroleum distillates present a higher risk of their children developing hypospadias (54) and present more allergic and non-allergic wheeze (55). A recent study identified a role of prenatal environmental and occupational exposures to endocrine disruptive chemicals in the development of hypospadias (56). The types of compounds involved were diverse, but detergents, pesticides, and cosmetics accounted for 75% of the cases of hypospadias. One should note that these are complex mixtures, which can contain adjuvants. In fact, the role of solvents in the toxicity of pesticides is well characterized, and most incidences of intoxication caused by organophosphorus pesticides can be attributed to their solvent content (57). Similarly, in cases of glyphosate-based herbicide exposure, the adjuvant content is known to be responsible for acute toxic effects (58). Thus, it is clear that adjuvants are responsible for most cases of acute toxicity of some commercial formulations of pesticides. The *in vitro* studies conducted by Séralini and colleagues using human cell culture model systems demonstrating far higher toxicity of commercial pesticide formulations, namely three insecticides (containing pirimicarb, imidacloprid, and acetamiprid), three fungicides (containing tebuconazole, epoxiconazole, and prochloraz), and three herbicides (containing glyphosate, isoproturon, and fluroxypyr) (59), corroborates these findings. Of the nine formulations tested, eight were up to one thousand times more toxic than their stated active principle. This was due to the presence of xylene, 1-methyl-2-pyrrolidinone, solvent naphtha, or *N*,*N*-dimethyldecanaminde, among other toxic compounds, in the adjuvant mixtures present in the pesticide formulations (59).

In typical chronic environmental exposures, when pesticide residues are found in tap water, food, or feed, they arise from the total formulation and not only from the active ingredients. Adjuvants are indeed found in groundwater. The total concentration of six alcohol ethoxylates was found to be 710 ng/L in the groundwater of one agricultural area (60). High concentrations of these compounds (10–190 mg/kg), as well as of nonylphenol (25–600 mg/kg), can be found in sewage sludge collected from treatment plants (61). Nonylphenol is of particular concern as it is a known endocrine disruptive chemical originating from surfactants and has been found to be involved in the widespread feminization of wild fish in UK rivers (62). However, little is known about the contamination of the environment, or even of human body fluids, by surfactants used as adjuvants in pesticides.

The exposure to molecules viewed as “inert” by regulators, but which are known to be toxic, is widespread and not limited to agricultural pesticides. This also includes cosmetics, drugs (including veterinary products), disinfectant products, and even food additives. In the United States, additives in food products are covered and regulated by the Food Additives Amendment passed by Congress in 1958 (63). This encompasses the status of substances that are “generally recognized as safe,” so they can be added to food without a review of safety by the Food and Drug Administration. This includes synthetic dyes or preservatives, which have been linked to negative health outcomes in humans (34, 64). Moreover, some chemicals are considered to be active ingredients in some products and inert adjuvants in others, with the distinction between “inert” and “active” being more of a regulatory question rather than a toxicology issue (65).

## Recommendations

The study of the effects of chemical mixtures on health indicators is frequently aired as a priority for the field of toxicology in the twenty-first century. However, within this framework, ignoring the toxicity of the combination of each active ingredient with its adjuvants could lead to misrepresentations of the safety profile of commercial pesticides. Therefore, we recommend the following actions to protect the public from toxicity that may arise from ingestion of adjuvants:

1. Biomonitoring of different human population groups to identify the true body burden of adjuvant classes of chemicals.
2. Surveying of food products to accurately identify sources of exposure.
3. Long-term laboratory animal toxicity studies comparing commercial formulations with their active principle to measure adverse outcomes stemming from the adjuvants.
4. The gaps in knowledge and consequent uncertainties in risk assessment concerning the toxicity of chemical mixtures, including adjuvants, need to be acknowledged by regulators. Thus, an additional safety factor needs to be added when calculating MRL and ADI values.
5. All ingredients used in the manufacture of commercial formulations of pesticides should be subjected to the same risk assessment. The classification as inert or active has no scientific basis.

Given the all-pervasive nature of adjuvants in products used in both an agricultural and urban/domestic environment, potential toxicity arising from exposure to these chemical mixtures can be greater than from any pesticide active principle. Although we are aware that all chemicals have intrinsic toxicological properties and that hazardous chemical properties do not necessarily translate into a risk for human health, it is scientifically not sound to argue that adjuvants are so safe that they can be ignored. The implementation of the above recommendations will allow major progress to be made in protecting the environment and general human population from these toxicants. Current practices in risk assessment and regulation fall far short of providing such protection.

## Author Contributions

All authors listed have made a substantial, direct, and intellectual contribution to the work and approved it for publication.

## Conflict of Interest Statement

The authors declare that the research was conducted in the absence of any commercial or financial relationships that could be construed as a potential conflict of interest.
